# Urogynaecological Symptoms among Oncological Survivors and Impact of Oncological Treatment on Pelvic Floor Disorders and Lower Urinary Tract Symptoms. A Six-Month Follow-Up Study

**DOI:** 10.3390/jcm9092804

**Published:** 2020-08-30

**Authors:** Alicja Ziętek-Strobl, Konrad Futyma, Izabela Kuna-Broniowska, Małgorzata Wojtaś, Tomasz Rechberger

**Affiliations:** 1 2nd Department of Gynecology, Medical University of Lublin, Jaczewskiego 8, 20-954 Lublin, Poland; futymakonrad@mp.pl (K.F.); malgorzata.woytas@gmail.com (M.W.); rechbergt@yahoo.com (T.R.); 2 Department of Applied Mathematics and Computer Science, University of Life Science, Akademicka 13, 20-950 Lublin, Poland; izabela.kuna@up.lublin.pl

**Keywords:** gynaecological malignancy, endometrial cancer, cervical, cancer, ovarian cancer, pelvic floor dysfunctions, lower urinary tract symptoms, quality of life, urogynaecological counseling

## Abstract

It has been widely underlined that both gynaecological malignancies and urogynaecological disorders are often associated with high stress and have a negative impact on the quality of life and psychological well-being of women affected. Knowledge of the pelvic anatomy is crucial in recommending and carrying out the least harmful although successful treatment. Subsequent chemoradiation may also induce or exaggerate troublesome symptoms. The aim of the study was to establish the frequency of urogynaecological symptoms (stress urinary incontinence, urgency, pelvic organ prolapse) and to assess the impact of surgical treatment and additional oncological therapy: pelvic radiation, chemoradiation, chemotherapy, on the prevalence of pelvic floor dysfunctions (PFD) and lower urinary tract symptoms (LUTS) in patients suffering from gynecological malignancies. The study group consisted of 160 women, diagnosed with gynaecological malignancy, who underwent surgical treatment and additional adjuvant treatment as necessary. To establish the QoL and prevalence of PFD Urinary Distress Inventory-6 (UDI-6), Incontinence Impact Questionnaire 7 (II-Q7), King’s Health Questionnaire (KHQ) and the SF-36 Questionnaire were used. Herein, 69 patients reported urinary incontinence (UI) and 67 reported symptoms of pelvic organ prolapse (POP). After the six months follow-up UI was found in 78 patients, 25 patients showed de novo symptoms, 65 patients reported POP and 10 patients demonstrated de novo POP. Our data show that urogynaecological symptoms are not correlated with the type of malignancy, but with the extensiveness of surgery.

## 1. Introduction

The common embryological, topographic and functional origin of the genitourinary tract implies also the potential being present for collective adverse reactions during or after management of oncological condition [1]. Apart from the fact that knowledge of the anatomy and topography of the genitourinary tract is crucial in recommending and carrying out the least harmful although successful treatment, it must be kept in mind that subsequent radio-chemotherapy may also induce or exaggerate troublesome symptoms [2,3]. Furthermore, oncological therapy can lead to adverse effects of long-term related treatment that often significantly and negatively affect the quality of life (QoL) among female patients. Considering the genitourinary tract, worldwide demographic trends lead to the conclusion that morbidity and mortality related to malignant conditions will increase the burden of post-treatment pelvic floor disorders (PFD) and lower urinary tract symptoms (LUTS) [4]. This is also confirmed by the International Oncological Committee, which claims that the trend of incidence of gynecological malignancies among young women (20–44 years of age) is increasing systematically [5]. Although PFD and LUTS are common among oncological patients, both still remain underestimated and not properly expostulated by patients and health care providers [6,7]. Moreover, both LUTS and PFD are associated with lower QoL, worse psycho-social functioning and emotional problems [8,9,10]. An international classification includes more than 100 malignant diseases, but studies on etiology, pathogenesis and treatment are limited to those occurring most frequently [1]. Nevertheless, the research on PFD and LUTS among oncological survivors still remains very limited. The primary aim of the study was to establish the frequency of urogynaecological symptoms among female oncological patients suffering from gynecological malignancies. A secondary aim was to assess the impact of surgical treatment and additional oncological therapy on the prevalence of PFD and LUTS in oncological survivors at six months follow-up.

## 2. Materials and Methods

**Figure 1:**
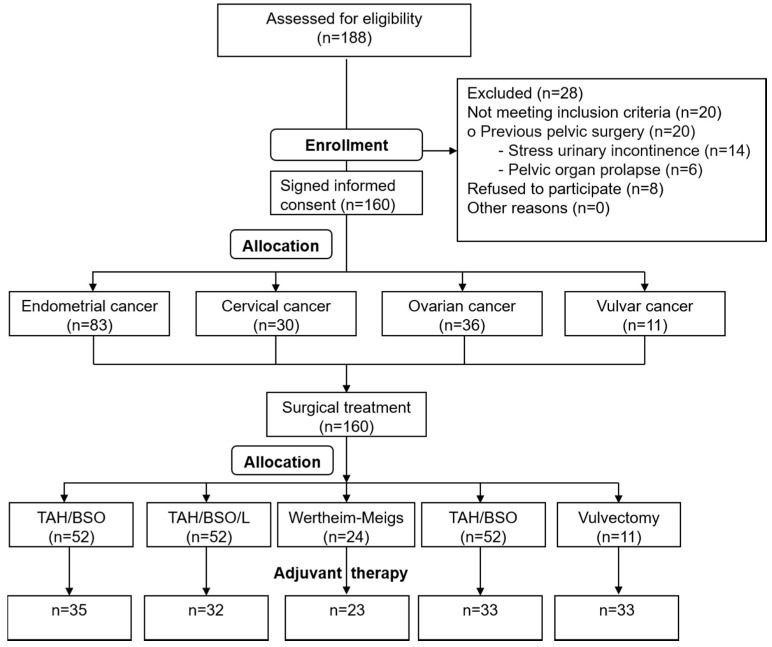
Study enrollment criteria.

This is a retrospective study. The study group consisted of 160 women, aged 28–87 years, who were diagnosed with gynaecological malignancy, and had undergone surgical treatment in the tertiary gynecological department between July 2015–March 2016. The study was approved by the Ethical Committee of Medical University (Ethical Approval No. KE-0254/206/2017). The enrolled patients were diagnosed with: endometrial cancer (E), cervical cancer (C), ovarian cancer (O) and vulvar cancer (V). Flow chart of participants is shown in Figure 1.

Before enrolment, all patients signed informed consent and agreed to use their data for scientific purposes. Inclusion criteria were as follows: confirmed cancer diagnosis, age more than 18 years old, cognitive and communication clarity, and awareness of the oncological diagnosis. None of the eligible patients had undergone previous operations due to pelvic organ disorders. The patients were asked to self-assess subjectively using specific questionnaires: the QoL, LUTS: i.e., storage, voiding and post-micturition symptoms and pelvic organ prolapse, defined as “vaginal bulge”, prior to and 6 months after the oncological treatment. Complete outcomes of 160 participants were collected in June 2017. The following questionnaires were used to establish the QoL and prevalence of PFD in the examined group: Urinary Distress Inventory-6 (UDI-6)—establishing the intensity of urinary incontinence; Incontinence Impact Questionnaire 7 (II-Q7)—assessing the impact of urinary incontinence on everyday functioning [11]; King’s Health Questionnaire (KHQ)—assessing the QoL by urinary incontinence concomitance [12,13]; and the SF-36 Questionnaire—assessing general QoL [14,15]. General discrimination analysis was conducted in order to evaluate which constant and qualitative predictors are crucial risk factors for PFD in female oncological patients. This method allows to find most relevant predictors for the occurrence of PFDs in the study group. The variables used to calculate the risk of PFD’s were age, parity, obesity and general internal diseases: arterial hypertension, diabetes type of malignancy and PFD occurrence prior the oncological diagnosis.

Moreover, patients evaluated subjectively their well-being by the seven grades Likert Scale.

Collected data was statistically analyzed using Statistica 10 (StatSoft, Cracow, Poland). Mean and variance of data were calculated in accordance with the methods of Hozo et al. [16]. The risk ratio (RR) with 95% confidence interval (CI) was calculated for data on dichotomous variables, and mean difference (MD) with 95% CI for continuous variables. Statistical significance was evaluated using non-parametric tests: Kruskall-Wallis, Shapiro-Wilk and Wilcoxon signed rank test, with significance set at *p* < 0.05. Heterogenous ranks among study groups were evaluated using the Chi^2^ test with significance set at *p* < 0.05. General discrimination analysis was conducted in order to evaluate which constant and qualitative predictors are crucial risk factors for PFD in female oncological patients.

## 3. Results

Mean age of the studied patients was 59.13 ± 12.12 years, mean menopausal age—49.41 ± 5.36 years, and mean BMI ratio 26.7 ± 5.56. Detailed demographic data are given in Table 1 and Table 2.

The use of correlation coefficient between the presence of PFD and nominal factors: type of malignancy, cancer stage, type of surgery and anterograde therapy, makes it possible to calculate the C-Pearson contingency factor and V-Cramer contingency in the study group. The values did not reveal any significant differences with *p* value > 0.05. To confirm introductory results, we also used an affinity analysis technique that discovers co-occurrence relationships among the same factors in the study group. The results of this analysis were by the low level of null hypothesis, meaning that revealed associations is not statistically significant.

To benefit from integration of the results the logistic regression analysis was conducted. The main limitation of this method is the presence of many variables in the study group and the small number of subjects in some groups.

However, the use of Chi-square test revealed some statistically significant correlations between the presence of PFD and: age, BMI score, diabetes, the type of malignancy. The values revealed the significant differences with *p* value > 0.05.

In our study, abdominal hysterectomy was performed in 114 patients, including 52 treated with simple, total abdominal hysterectomy with bilateral salpingoophorectomy (TAH/BSO) and 38 with hysterectomy supplemented with pelvic lymphadenectomy (TAH/BSO/L), while 24 patients underwent radical Wertheim-Meigs hysterectomy. Out of 36 patients diagnosed with ovarian cancer, 35 underwent debulking surgery only without hysterectomy. Postoperatively patients underwent individualized, adjuvant oncological therapy. The ANOVA Kruskal-Wallis test was used for the comparison of independent variables and serves as a non-parametric alternative for the one-way analysis of variance. The test is based on observation rank. When all the variables come from one population, we expect mean ranks in specific groups to be similar.

The use of ANOVA Kruskal-Wallis test does not require an equipotencial number of subjects in the groups. The test’s function model contains the group size, so it does not issue the results (Figure 1, Table 3).

Detailed data concerning urinary incontinence (UI) and pelvic organ prolapse (POP) symptoms prevalence in the study group prior and after oncological treatment are given in Table 4.

In the group of 78 women suffering from UI only 20 patients (25.6%) underwent surgery alone and 58 patients (74.4%) had adjuvant oncological treatment. In the group of 65 women with POP symptoms, 19 patients (29.2%) underwent surgery alone and 46 patients (70.8%) had adjuvant oncological treatment.

We have also found (by means of general discrimination analysis) a combination of factors determining the prevalence of PFDs prior to oncological treatment (strength of discriminatory function given in brackets): age (2.52), multiparity (2.27), arterial hypertension (1.18) and the diagnosis of ovarian cancer with concomitant ascites (0.76).

Moreover, the same analysis determined factors responsible for increased risk of PFD de novo occurrence after oncological treatment: arterial hypertension (3.91), diagnosis of endometrial cancer (3.37), diagnosis of cervical cancer (3.27), obesity (2.78).

Analysis of seven grades Likert scale (1—the worst QoL, 7—the best QoL) did not revealed any statistically significant differences between the study groups, neither before nor after oncological therapy. Nonetheless, analysis of the lower quartile of the Likert scale indicated that 25% of patients assessed their general health as having deteriorated after treatment. However, analysis of the upper quartile revealed that at least 50% of the patients claimed that their general health had improved after surgical and oncological therapy.

UDI-6 Questionnaire. The UDI-6 Questionnaire assessed in the study groups the impact of oncological treatment on the prevalence of urinary incontinence symptoms. Aggregated results of the UDI-6 Questionnaire revealed a statistically significant deterioration of urinary incontinence symptoms in the study group of oncological survivors (Table 5).

Moreover, statistical analysis of the UDI-6 Questionnaire conducted between patients divided into separate groups according to the mode of surgical treatment, revealed significant differences between all study groups, apart from radical Wertheim-Meigs hysterectomy and simple vulvectomy. The lack of statistically significant differences in these study groups was probably due to the small number of patients (Table 6).

II-Q7 Questionnaire. The II-Q7 Questionnaire assessed the impact of oncological treatment on the prevalence of urinary incontinence symptoms and the impact of lower urinary tract symptoms on everyday life. Aggregated results of the II-Q7 Questionnaire revealed a statistically significant deterioration of the symptoms of urinary incontinence and life functioning in the study group (Table 5). Assessment of the mode of surgical treatment on the results of the II-Q7 Questionnaire is presented in Table 6.

King’s Health Questionnaire. The KHQ questionnaire domains as rated before and after 6 months, as given in Table 7. Statistical analysis revealed significant differences in all domains of the KHQ, with the exception of the severity measure domain.

As shown in Table 8, statistically significant differences were found in the individual surgical groups. No statistically significant differences were found in the vulvectomy surgical group, which is probably a result of the small number of patients (*p* > 0.05).

It should be noted that some oncological treatment groups are represented by small number of patients, which requires caution when drawing conclusions.

Statistically significant deterioration of QoL was found in particular surgical groups between patients with surgical treatment only and patients with adjuvant oncological treatment. In the TAH/BSO group, significance was found in H, RL and PL domains (*p* < 0.05) when comparing between patients with surgical treatment only, and patients with adjuvant radiotherapy. In the TAH/BSO/L group, significance was found in H, RL, PL, SL, PR, E and S/E domains (*p* < 0.05) when comparing between patients with surgical treatment only, and patients with adjuvant radiotherapy. In the Wertheim-Meigs group, significance was found only in GH domain (*p* < 0.05) when comparing between patients with surgical treatment only, and patients with adjuvant chemoradiation. In the debulking surgery group, significance was found only in PL domain (*p* < 0.05) when comparing between patients with surgical treatment only, and patients with adjuvant radiotherapy and in SL and PR domains (*p* < 0.05) when comparing between patients with surgical treatment only, and patients with adjuvant chemoradiation. Chemotherapy alone did not negatively influence the QoL.

There were no statistically significant differences in the vulvectomy surgical group, which is probably a result of the small number of patients (*p* > 0.05).

SF-36 Questionnaire. The QoL of oncological survivors was lower than in the general population in the following domains measuring physical health: physical functioning, (PF), bodily pain (BP), and in the domains measuring mental health: vitality (VT), role-emotional (RE) and mental health (MH). The domains of the SF-36 Questionnaire are classified into two main categories: physical component summary (PCS) and mental component summary (MCS). Statistical analysis conducted between the operative groups revealed significant differences in the MCS category only in the group of patients who underwent radical Wertheim-Meigs hysterectomy. This highlights the negative impact of extended damage to surrounding tissues, including vessels and innervation, which finally leads to the deterioration of mental health. Another statistical analysis was performed to assess the impact of additional oncological therapy. It was found that adjuvant oncological therapy did not significantly impact general and mental health after six months follow-up.

There were no statistically significant differences in UDI 6, II-Q7 and SF-36 Questionnaire results between patients with surgical treatment only, and patients with adjuvant oncological therapy (*p* > 0.05).

## 4. Discussion

Nowadays, thanks to the undoubted progress in clinical practice, demographic trends and availability of health care, therapeutic options should provide not only effective oncological therapies, but also an appropriate QoL for the population of oncological survivors. Elderly women comprise the majority group of urogynecological and oncological patients. Health care providers should be aware that treatment of gynaecological malignancy should not abandon lower urinary tract symptoms [17]. Undeniably, life prolongation of oncological survivors who often underwent radical treatment due to gynaecological malignancy, implicates an increase of iatrogenic complications, including PFD. Consequently, because these symptoms significantly deteriorate the QoL and treatment cost-effectiveness, they should be a matter of concern for healthcare professionals [18].

Our study reveals that symptomatic POP is a frequent comorbidity among oncological patients, especially when coexisting with such co-factors as older age, obesity, hypertension, diabetes and multiparity. Previously existing asymptomatic PFD are also a strong risk factor of de novo clinically significant prolapse after therapy. This finding is indeed an important argument in favor of using preventive procedures against POP (if plausible), while performing oncological surgeries. Urinary incontinence frequently coexists with POP, which has been confirmed by many studies [19]. In the study conducted by Bai et al., 63% of all patients treated for stress urinary incontinence suffered from coexisting pelvic organ prolapse. Additionally, 62% of all patients who underwent surgery due to POP also suffered from urinary incontinence [20].

The study results are coherent with the review presented by Ramaseshan et al. in 2018, which claimed that the prevalence of PFD has broad range of gynaecolgical cancer survivors. Although the limitation of both studies is the small number of vulvar cancer group [7].

Our study confirms results published by Donovan et al., who reported that urinary incontinence among gynaecological oncological survivors is not only frequent, but also significantly decreases the general QoL similarly or even worse, to non-oncological counterparts [21]. The survivors were significantly (*p* < 0.05) more likely to have symptoms of incontinence of every type. Survivors also reported significantly (*p* < 0.0001) lower QoL associated with these symptoms. The results obtained from the UDI-6 and II-Q7 questionnaires in our study reaffirmed that comprehensive oncological therapy impacts negatively on LUTS. These results are compatible with conclusions reported by Erekson et al. [22] who found that prevalence of urinary symptoms in a population of women treated for endometrial cancer was as high as 83.6%. In addition, women treated with adjuvant radiation therapy reported more severe incontinence symptoms and a higher impact on QoL. Analysis of the King’s Health Questionnaire confirmed that in the group of female oncological patients, the presence of urinary incontinence decreased general health and the QoL, although not in every domain. The most negative impact of oncological treatment, which was statistically significant, was found in the domains: role limitation, social limitation, emotions and sleep/energy.

The results of our study clearly show that surgery followed by pelvic radiation and chemoradiation had the most negative impact on PFD and QoL. This is consistent with results obtained by Erekson et al. and Khrut et al. [22,23]. Minimal incontinence was associated with a significant negative impact on QoL as measured by all QoL assessment tools. There were non-linear correlations between scores of KHQ on individual questionnaires. Furthermore, the mean questionnaire scores for women treated with adjuvant radiation therapy were higher compared to women with no adjuvant radiation therapy (47 ± 26.8 vs. 35.6 ± 21.7; *p* = 0.05) [22]. Abdominal hysterectomy with bilateral salpingoophorectomy remains the most frequently performed procedure among gynaecological oncological patients [23]. When necessary, it is extended by pelvic lymphadenectomy, paracolpium removal or debulking surgery. This more aggressive and radical surgery results in direct intra-operative and early post-operative complications and injuries to adjacent tissues, and participates in increasing the probability of urogynaecological symptoms [20,24,25,26]. Radical pelvic surgery aims to reduce the recurrence of the malignancy, but simultaneously may damage urogenital nerves and blood vessels. Tissue hypoxia, the scarring process and local ischemia can increase the probability of PFD. In the literature, there has been a growing trend to report mortality and morbidity after surgery with pre-operative risk adjustment to facilitate meaningful comparisons of surgical outcomes. The metanalysis conducted by Kim et al. reported that “nerve sparing” procedures, although longer-lasting, saves vessels and autonomic nerve plexuses, which statistically decreases the frequency of pelvic floor injuries and dysfunctions [27]. The results of this study lead to the conclusion that isolated surgical treatment implicates pelvic floor disorders; nonetheless, additional isolated oncological therapy does not significantly affect urogynaecological symptoms. However, the combined treatment-surgery plus radiation and chemoradiation, negatively affects the lower urinary tract and organs of the pelvic floor. The risk of complications after pelvic radiation is undeniable, impacting not only on the genitourinary, but also the alimentary tract. This can affect patients directly or for a long time after the treatment, and include: urgency, stress urinary incontinence, interstitial cystitis, urinary bladder fibrosis, vaginal fibrosis and vesico/urethro-vaginal fistulas [25]. In one of our patients with cervical cancer (FIGO stage Ia) who underwent Wertheim-Meigs’ surgery with adjuvant chemoradiation, vesico-vaginal fistula occurred three months after therapy completion. There is no consensus in the literature concerning the impact of pelvic radiation on lower urinary tract symptoms or organ prolapse. Erekson et al. and Manchana et al. have stated that the use of radiation decreases pelvic floor function and the QoL of oncological survivors, whereas the results of other studies contradict these reports [22,25,26,27,28]. The PORTEC-2 study conducted by de Boer et al. on a group of 427 female oncological survivors revealed that in the seven and 10-years observation time, the frequency of lower urinary tract symptoms was higher, although it did not negatively affect the QoL of the patients [29]. This correlates with results presented by Pisani et al., who conducted a study on a group of endometrial and cervical cancer patients [30].

In our study, the results of the SF-36 Questionnaire showed that the quality of both the physical and the mental life of female oncological patients were lower than in the general population. However, in analyzing isolated surgery and adjuvant oncological therapy, no statistically significant differences were found. MCS was significantly decreased in the group of patients who underwent radical hysterectomy accompanied by chemoradiation. This complies with a study by White et al., on a group of 9282 oncological patients diagnosed with endometrial, breast, colon, lung, prostate and urinary bladder cancers. They reported that surgery followed by oncological treatment raised the prevalence of mental health disorders, such as depression, dysthymia and neurosis [31]. The QoL of female oncological survivors is significantly decreased by the presence of pelvic floor disorders. Clinical practice and therapeutic methods should take these symptoms into consideration and offer preventive techniques and appropriate management to the patients.

The limitations of our study are its relatively small number of patients with each type of cancer and categorized into the sub-treatment groups. Especially, the significant differences between the participants numbers assigned to sub treatments limits the ability to clearly state which mode of treatment increases the risk of post-treatment PFDs. Another limit is lack of validated scales assessing the severity of UI or POP.

The advantage of our study is that physicians will be made aware of urogynaecological symptoms that may coexist with diagnosed malignancy and deteriorate the QoL even more than the cancer itself. It should be noted that gynecological oncologists are rarely thinking in that way. Moreover, it might be helpful in counseling oncological patients and preparing them for complications or typical adverse events that might be associated with the treatment patient is going to go through. This is a very important issue, and surgeons should seek to accomplish all tasks necessary for a shared communication model [7,32].

## 5. Conclusions

More than 50% of all females diagnosed with a gynaecological malignancy suffered from PFD or UI at the time of oncological diagnosis.

As treatment techniques improve overall survival of patients, the influence of therapy on everyday functioning is an important aspect of pretreatment counseling.

Urogynaecological symptoms are correlated with the mode of treatment applied and not with the type of malignancy. Surgical treatment alone is a major risk factor of QoL worsening, due to an increase in urinary incontinence and its negative impact on everyday functioning. Furthermore, combined therapy especially radiotherapy or chemoradiation, might have additional negative influence on particular domains of general QoL. Lastly, adjuvant chemotherapy does not deteriorate PFD and UI symptoms thus it does not negatively influence QoL—which is crucial especially for patients with ovarian cancer.

## Figures and Tables

**Table 1:** Stratification of patients according to type of gynaecological malignancy and age.

Age Groups	E	C	O	V
W1	14	10	15	2
W2	27	8	6	2
W3	18	8	9	2
W4	24	4	6	5
Total	83	30	36	11

W1—28–50 years of age; W2—51–60 years of age; W3—61–67 years of age; W4—>68 years of age; E—endometrial cancer; C—cervical cancer; O—ovarian cancer, V—vulvar cancer.

**Table 2:** Patients’ characteristics according to FIGO staging and type of malignancy considering concomitance of urinary incontinence and pelvic organ prolapse.

FIGO	E				C				O				V			
		UI	POP	UI + POP		UI	POP	UI + POP		UI	POP	UI + POP		UI	POP	UI + POP
I	68	23 (33.8%)	30 (44.1%)	19 (27.9%)	23	14 (60.9%)	10 (43.5%)	9 (39.1%)	9	6 (66.7%)	5 (55.6%)	4 (44.4%)	7	1	-	-
II	13	9 (69.2%)	11 (84.6%)	7 (53.8%)	6	2 (33.3%)	2 (33.3%)	1 (16.7%)	12	9 (75%)	7 (58.3%)	5 (41.7%)	4	-	-	-
III	2	1 (50%)	-	1 (50%)	1	-	-	-	13	3 (23.1%)	2 (15.4%)	1 (7.7%)	0	-	-	-
IV	0	-	-		0	-	-	-	2	1 (50%)	-	-	0	-	-	-
Total	83	33 (39.8%)	41 (49.4%)	27 (32.5%)	30	16 (53.3%)	12 (40%)	10 (33.3%)	36	19 (52.8%)	14 (38.9%)	10 (27.8%)	11	1	-	-

FIGO: International Federation of Gynecology and Obstetrics (Fédération Internationale de Gynécologie et d’Obstétrique).

**Table 3:** Anterograde oncological treatment in study groups.

Type of Surgical Treatment	Radiation (*n*)	Chemoradiation (*n*)	Chemotherapy (*n*)	None (*n*)
TAH/BSO (*n* = 52)	29	4	2	17
TAH/BSO/L (*n* = 38)	16	13	3	6
Wertheim-Meigs hysterectomy (*n* = 24)	1	18	4	1
Debulking surgery (*n* = 35)	6	4	23	2
Vulvectomy (*n* = 11)	6	3	0	2

**Table 4:** Urinary incontinence and POP symptoms prevalence in the study group prior and after oncological treatment.

	Before *n* (%)	At Follow-Up			
		After *n* (%)	De Novo *n* (%)	Reduction of Symptoms *n* (%)	Resolution of Symptoms *n* (%)
Urinary incontinence (UI)	69 (43.0%)	78 (48.8%)	25 (32.1%)	5 (6.4%)	16 (20.5%)
Concomitant POP	40 (58.0%)	49 (62.8%)	14 (28.6%)	2 (4%)	5 (10.2%)
Pelvic organ prolapse (POP)	67 (41.9%)	65 (40.6%)	10 (15.4%)	2 (3.1%)	12 (18.4%)
Concomitant UI	44 (65.7%)	32 (49.2%)	6 (18.8%)	1 (3.1%)	18 (56.3%)

**Table 5:** Impact of oncological treatment on the prevalence of urinary incontinence symptoms and impact on patients’ everyday life (UDI-6 and II-Q7 questionnaires) (Wilcoxon’s signed-rank test).

Questionnaire	*n*	Mean	SD	T	Z	*p*
UDI-6 before	160	38.64	24.11	1689.00	4.69	*p* < 0.05
UDI-6 after		51.04	28.47			
II-Q7 before	160	54.02	27.59	2373.00	6.93	*p* < 0.05
II-Q7 after		76.79	36.62			

**Table 6:** Impact of the mode of surgical treatment performed on the prevalence of urinary incontinence symptoms and impact on patients’ everyday life (UDI-6 and II-Q7 questionnaires) (Wilcoxon’s signed-rank test).

Questionnaire		UDI-6					II-Q7				
		Before	After				Before	After			
Surgery Type	*n*	Mean (±SD)	Mean (±SD)	T	Z	*p*	Mean (±SD)	Mean (±SD)	T	Z	*p*
TAH/BSO	52	34.21 (22.83)	47.67 (27.2)	141	3.48	*p* < 0.05	47.25 (18.28)	71.15 (30.42)	132.5	5.07	*p* < 0.05
TAH/BSO/lymphadenectomy	38	41.23 (26.93)	54.17 (26.88)	75.5	2.73	*p* < 0.05	59.4 (32.61)	82.46 (39.36)	180	2.76	*p* < 0.05
Wertheim-Meigs radical hysterectomy	24	41.67 (23.79)	50.17 (32.5)	46	1.44	*p* < 0.05	52.98 (29.4)	79.56 (40.61)	39	3.17	*p* < 0.05
Debulking surgery	35	38.57 (23.45)	53.93 (32.18)	73.5	2.39	*p* < 0.05	55.92 (31.47)	80.68 (41.03)	129	3.05	*p* < 0.05
Vulvectomy	11	48.86 (22.75)	48.86 (19.01)	18	0	*p* < 0.05	63.64 (25.4)	65.37 (29.21)	27	0.53	*p* < 0.05

**Table 7:** King’s Health Questionnaire (KHQ) results in the study group before surgery and after 6 months follow-up (Wilcoxon’s signed-rank test).

	Before	After		
Domain	Mean (±SD)	Mean (±SD)	Z	*p*
GH	41.87 (21.99)	51.17 (28.17)	2.13	*p* < 0.05
II	35.41 (23.55)	50.31 (34.75)	3.96	*p* < 0.05
RL	38.12 (26.12)	51.15 (30.98)	4.02	*p* < 0.05
PL	36.45 (22.28)	50.1 (28.75)	10.93	*p* < 0.05
SL	33.47 (2.9)	48.21 (31.44)	3.67	*p* < 0.05
PR	28.33 (30.47)	44.02 (41.22)	3.55	*p* < 0.05
E	30.76 (26.26)	46.89 (35.37)	10.60	*p* < 0.05
S/E	53.77 (38.24)	53.77 (38.24)	4.06	*p* < 0.05
SM	51.91 (31.06)	57.94 (35.16)	1.62	*p* < 0.05

GH—general health; II—incontinence impact; RL—role limitation; PL—physical limitation; SL—social limitation; PR—personal relationship; E—emotions; S/E—sleep/energy; SM—severity measure.

**Table 8:** Impact of the mode of surgical treatment performed on the prevalence of urinary incontinence symptoms and impact on patients’ everyday life measured by the various KHQ domains (Wilcoxon’s signed-rank test).

Surgery Type	Domain								
	GH	II	RL	PL	SL	PR	E	S/E	SM
TAH/BSO		*p* < 0.05	*p* < 0.05	*p* < 0.05	*p* < 0.05			*p* < 0.05	
TAH/BSO/lymphadenectomy	*p* < 0.05	*p* < 0.05	*p* < 0.05	*p* < 0.05	*p* < 0.05		*p* < 0.05	*p* < 0.05	
Wertheim-Meigs radical hysterectomy			*p* < 0.05	*p* < 0.05	*p* < 0.05	*p* < 0.05	*p* < 0.05	*p* < 0.05	
Debulking surgery			*p* < 0.05	*p* < 0.05	*p* < 0.05	*p* < 0.05	*p* < 0.05	*p* < 0.05	
Vulvectomy									

Empty slot means *p* value > 0.05.
